# Severe Neurovascular Compromise Associated with Plastic Deformation of Ulna Elastic Nail Following Forearm Re-fracture

**DOI:** 10.7759/cureus.4340

**Published:** 2019-03-28

**Authors:** Zakk M Borton, Simon Weil, Edward F Ibrahim, Callum Clark

**Affiliations:** 1 Trauma and Orthopaedics, Royal Derby Hospital, Derby, GBR; 2 Trauma and Orthopaedics, St. Georges University, London, GBR; 3 Trauma and Orthopaedics, West Middlesex University Hospital, London, GBR; 4 Trauma and Orthopaedics, Frimley Park Hospital NHS Foundation Trust, Frimley, GBR

**Keywords:** flexible intramedullary nailing, trauma, paediatric forearm fractures, elastic intramedullary nails, fracture

## Abstract

We present a case of periprosthetic re-fracture of the forearm in a child with previous intramedullary elastic nailing of the ulna and plate fixation of the radius for a both-bone forearm fracture. In-situ plastic deformation of the ulna elastic nail resulted in persistent angulation and subsequent severe neurovascular compromise. The angulation was resistant to emergent attempts at closed manipulation and therefore nail removal, open reduction, and internal fixation were performed. At final follow-up, fracture union was demonstrated and there was no residual neurological deficit.

## Introduction

Elastic stable intramedullary nailing (ESIN) and plate fixation are recognized strategies for operative management of displaced pediatric diaphyseal forearm fractures. Management strategy depends on fracture configuration and post-reduction deformity, amongst other factors such as the age and skeletal maturity of the child. There is no definitive consensus on whether plate or ESIN fixation is superior, with recent studies reporting no statistical difference between the two with regard to functional and radiological outcome [1]. Re-fracture is a recognized complication [2-3]. An increasing tendency towards operative management of these fractures has been reported and as such, it is likely that the incidence of re-fracture with metalwork in-situ will also rise [4].

We present a case of periprosthetic re-fracture in a 13-year-old male following ulna intramedullary (IM) elastic nailing and radial plating. Severe angular deformity with the subsequent neurovascular compromise of the forearm was maintained secondary to in-situ plastic deformation of the ulna nail. This case raises unique questions regarding the management of this injury and highlights possible implications regarding mixed-modality operative management of pediatric forearm fracture.

## Case presentation

A 13-year-old Caucasian male sustained diaphyseal fractures of the left radius and ulna three months prior to the presentation in focus. The fractures were managed surgically with closed reduction and 3.0 mm intramedullary elastic nailing of the ulna and open reduction and internal fixation (ORIF) of the radius with a 3.5 mm dynamic compression plate (DCP). Follow-up at two months confirmed satisfactory progress and radiological evidence of the union. The patient was scheduled for elective removal of the elastic nail at three months.

**Figure 1:**
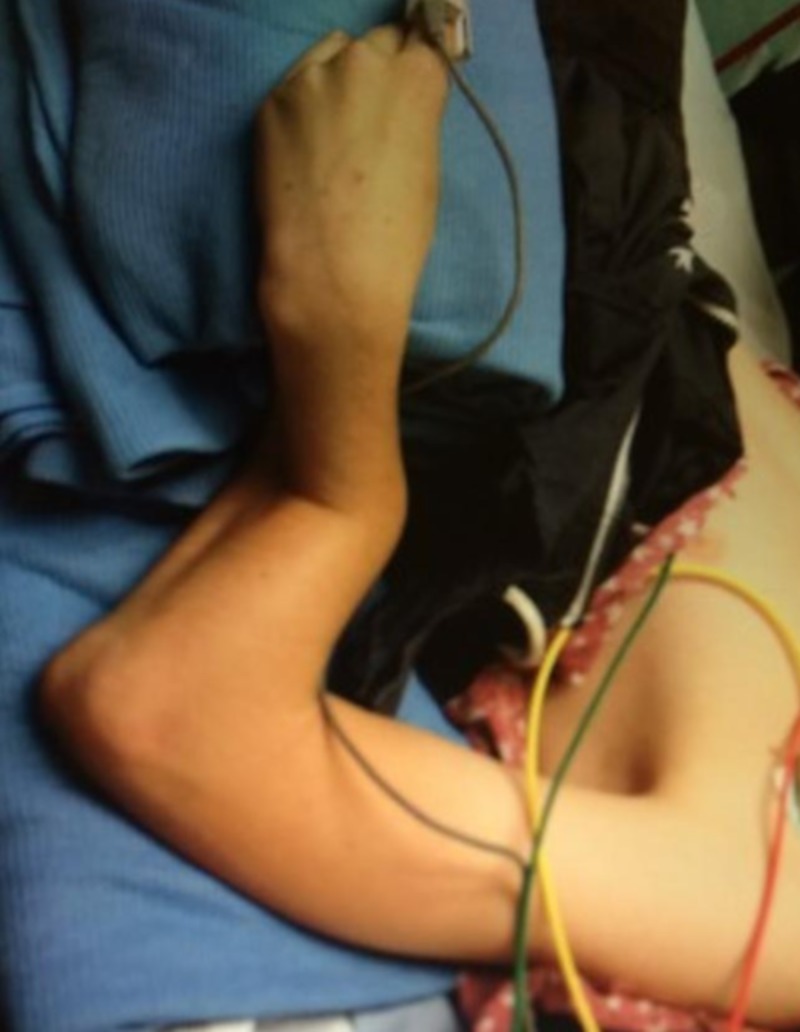
Clinical photograph of the affected limb on presentation The forearm is severely angulated and pallor can be seen in the hand. The oxygen saturation probe placed on the finger was unable to register a signal due to vascular compromise.

One week prior to metalwork removal the patient tripped and fell onto his outstretched left hand. He presented with severe clinical deformity of the forearm and severe distal neurovascular deficit (Figure 1). The hand was pale with an absent radial pulse, prolonged capillary refill time (CRT) and no digital signal on pulse oximetry. Furthermore, both sensory numbness and motor weakness in the distributions of both median and ulna nerves were noted.

**Figure 2:**
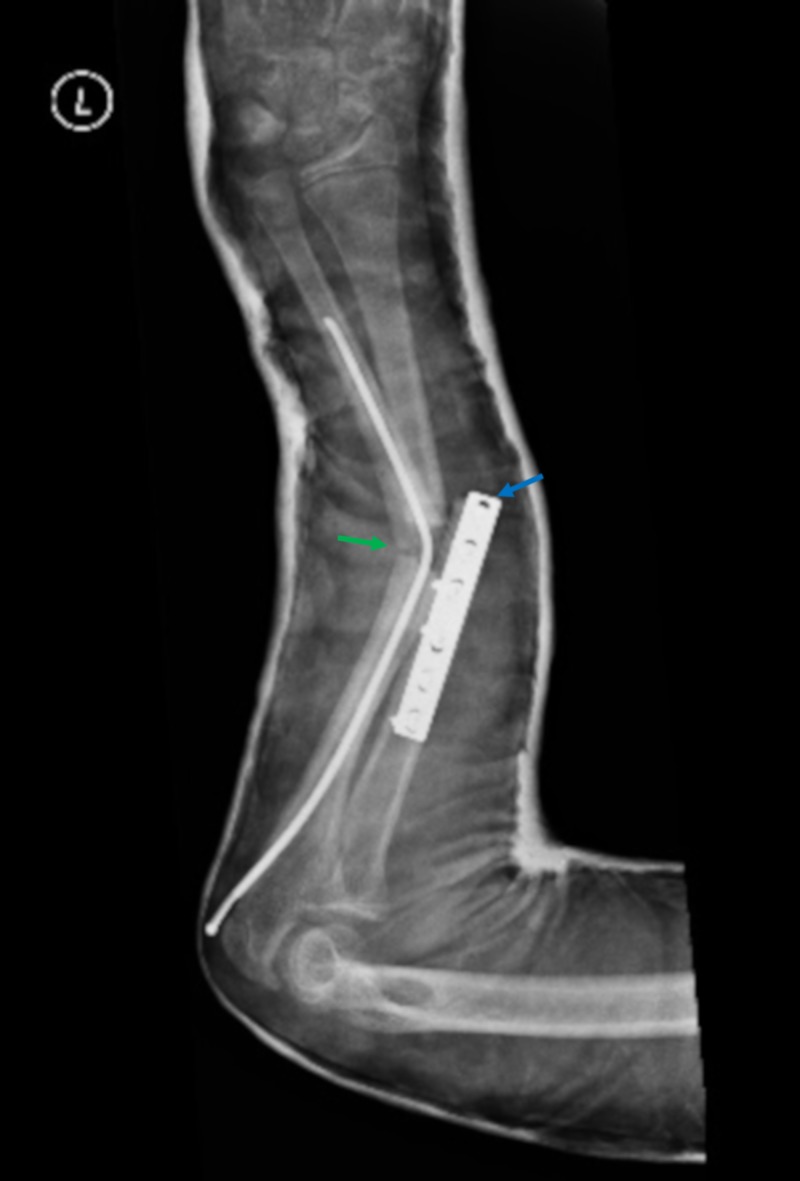
Presenting radiograph Single view radiograph demonstrating plastic deformation of the elastic stable intramedullary nailing in the context of ulna re-fracture (green arrow) and a new fracture at the distal margin of the radial plate (blue arrow).

Emergent closed reduction in the emergency department was only partially successful in restoring alignment, with restoration of the CRT but persistent neurological deficit. Post-reduction radiographs revealed persistent angular deformity of sixty degrees. Re-fracture of the ulna with plastic deformation of the flexible nail was again noted, as was a periprosthetic fracture at the distal margin of the radial plate (Figure 2).

In view of the persisting neurological deficit, the patient was taken to the operating theater expediently. Further attempts at closed manipulation under general anesthesia were unsuccessful. The IM nail- though deformed- was intact allowing successful removal via the routine method from the proximal ulna entry point; however, satisfactory reduction could still not be gained. The radius was approached through the previous volar approach and re-plated, ensuring use of a longer 3.5 mm DCP to account for the previous screw holes. The ulna was then approached directly and internal fixation with a second 3.5 mm DCP performed. Postoperative recovery was untoward and the patient was discharged after overnight observation.

**Figure 3:**
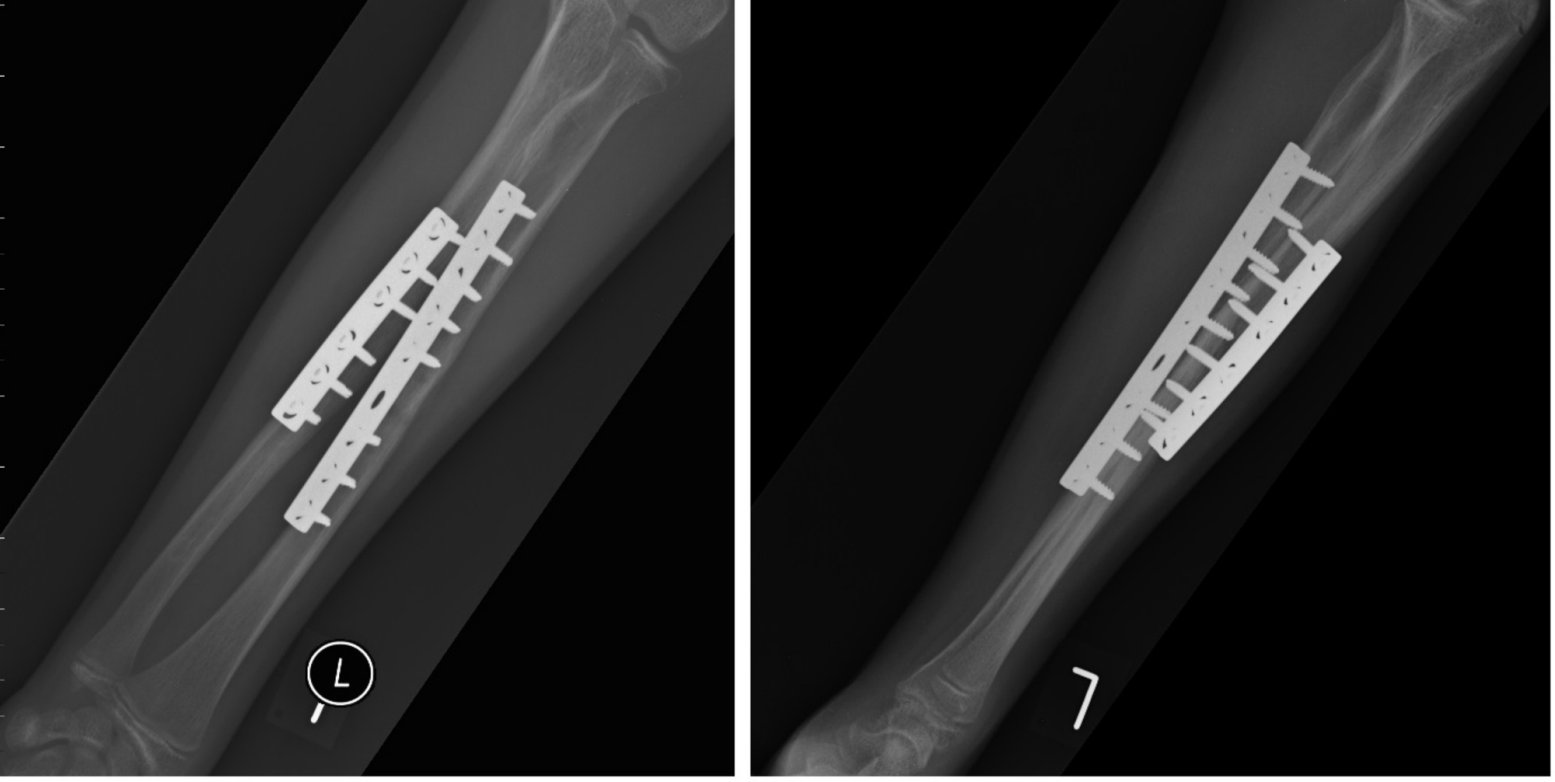
Postoperative radiographs Orthogonal radiographs of the forearm at two month follow-up visit showing union of both bones with no evidence of synostosis.

At two weeks follow-up there was complete resolution of both subjective and objective neurological deficits. Early active forearm rotation and elbow flexion were commenced at this stage with splinting of the wrist for comfort. At two-month follow-up, the radiographs revealed fracture union (Figure 3).

## Discussion

We have identified a number of case reports and small case series reporting on re-fracture subsequent to flexible nailing of pediatric forearm fractures (time to re-fracture 1-45 months, patient age 4-16 years) [5-10]. To our knowledge, our reported case is novel with respect to the combination of modalities at primary surgery, with ESIN of the ulna and plate fixation of the radius. In all of the above cases, the flexible nails did not break but instead deformed to varying degrees of angulation (ranging from 20-80 degrees). All re-fractures occurred at the initial fracture site and none resulted in a neurovascular compromise on presentation, both of which highlight further areas in which our own case is novel to the literature.

Several treatment options have been described, with the most common being closed reduction and casting. Other modalities include the exchange of the nails or nail removal with subsequent ORIF. Shahid et al. also reported successful management with closed reduction and partial retraction of the radial nail. All reported satisfactory results at follow-up. In our case closed reduction was unsuccessful, necessitating removal of metalwork and re-fixation for both radius and ulna. ESIN fixation of both the radius and the ulna would have been an alternative method of fixation, though difficulty with closed reduction and concern regarding the challenge of passing the nail across the healed radial primary fracture site influenced the decision to proceed to plate fixation of both fractures.

Proponents of hybrid fixation have published small series and biomechanical studies citing equivalent union rates, time to union, and biomechanical stability with improved operative time, fluoroscopy time, and cosmesis versus both-bone IM nailing or both-bone ORIF [11-14]. We postulate that the hybrid implant combination presented in our case could have contributed to the severe deformity and neurovascular compromise on presentation. Biomechanically, it follows that if flexible intramedullary nails had been employed for both bones at the time of primary surgery, then the greater stiffness and load-sharing between implants may have led to less angulation for any given impulse. Conversely, if two plates had been used then the natural elastic recoil usually seen with forearm fractures would likely have occurred, preventing the sustained severe angulation caused by the deformed nail.

## Conclusions

We present a rare case of sustained angulation caused by in-situ plastic deformation of an intramedullary nail following re-fracture with subsequent neurovascular compromise. Clinicians utilizing hybrid or IM fixation strategies should be cognisant of this potential sequela. The evidence base informing the optimum management of pediatric forearm fractures is heterogeneous and lacks high-level studies. The highly powered randomized studies and meta-analyses are required to better elucidate the optimum management of these fractures.

## References

[1] Baldwin K, Morrison MJ, Tomlinson LA, Ramirez R, Flynn JM (2014). Both bone forearm fractures in children and adolescents, which fixation strategy is superior - plates or nails? A systematic review and meta-analysis of observational studies. J Orthop Trauma.

[2] Martus JE, Preston RK, Schoenecker JG, Lovejoy SA, Green NE, Mencio GA (2013). Complications and outcomes of diaphyseal forearm fracture intramedullary nailing: a comparison of pediatric and adolescent age groups. J Pediatr Orthop.

[3] Flynn JM, Jones KJ, Garner MR, Goebel J (2010). Eleven years experience in the operative management of pediatric forearm fractures. J Pediatr Orthop.

[4] Schmittenbecher PP (2005). State-of-the-art treatment of forearm shaft fractures. Injury.

[5] Mittal R, Hafez MA, Templeton PA (2004). Failure of forearm intramedullary elastic nails. Injury.

[6] Changulani M, Garg NK, Bruce CE (2005). Use of ESIN in forearm fractures in children: does keeping the nail in situ longer prevent refractures. Injury Extra.

[7] Shahid M, Yeo M, Smibert JG (2011). Closed reduction of radius refracture: a case report. Int J Surg Case Rep.

[8] van Egmond PW, van der Sluijs HA, van Royen BJ, Saouti R (2013). Refractures of the paediatric forearm with the intramedullary nail in situ. BMJ Case Rep.

[9] Muensterer OJ, Regauer MP (2003). Closed reduction of forearm refractures with flexible intramedullary nails in situ. JBJS.

[10] Kelly BA, Shore BJ, Bae DS, Hedequist DJ, Glotzbecker MP (2016). Pediatric forearm fractures with in situ intramedullary implants. J Child Orthop.

[11] Cai L, Wang J, Du S, Zhu S, Wang T, Lu D, Chen H (2016). Comparison of hybrid fixation to dual plating for both-bone forearm fractures in older children. Am J Ther.

[12] Behnke N, Redjal H, Nguyen V, Zinar D (2012). Internal fixation of diaphyseal fractures of the forearm: a retrospective comparison of hybrid fixation versus dual plating. J Orthop Trauma.

[13] Zhang XF, Huang JW, Mao HX, Chen WB, Luo Y (2016). Adult diaphyseal both-bone forearm fractures: a clinical and biomechanical comparison of four different fixations. Orthop Traumatol Surg Res.

[14] Feng Y, Shui X, Wang J, Cai L, Wang G, Hong J (2016). Comparison of hybrid fixation versus dual intramedullary nailing fixation for forearm fractures in older children: case-control study. Int J Surg.

